# Efficacy and safety of Shufeng Huashi formula for post-infectious cough: study protocol for a randomized control trial

**DOI:** 10.3389/fmed.2026.1821956

**Published:** 2026-06-01

**Authors:** Xinyi Zhang, Yirun Li, Yuxin Han, Hongchun Zhang

**Affiliations:** 1 Graduate School, Beijing University of Chinese Medicine, Beijing, China; 2 National Center for Respiratory Medicine; State Key Laboratory of Respiratory Health and Multimorbidity; National Clinical Research Center for Respiratory Diseases; Institute of Respiratory Medicine, Chinese Academy of Medical Sciences; The First Department of Integrative Pulmonology, Center of Respiratory Medicine, China-Japan Friendship Hospital, Beijing, China

**Keywords:** post-infectious cough, randomized controlled trial, Shufeng Huashi formula, study protocol, Traditional Chinese Medicine

## Abstract

**Background:**

Post-infectious cough (PIC) refers to a type of persistent cough triggered by respiratory tract infections that remains unresolved after the initial infection is controlled. It significantly impacts daily life, work, and overall quality of life. This study aims to evaluate the clinical efficacy and safety of the Shufeng Huashi (SFHS) formula, a Traditional Chinese Medicine (TCM) compound, in patients with PIC.

**Methods and analysis:**

This multicenter, randomized, double-blind, placebo-controlled, parallel-group clinical trial will recruit 120 patients aged 18–65 who meet the diagnostic criteria for PIC. Participants will be randomly assigned to the SFHS group or the placebo group in a 2:1 ratio using a random coding table. The SFHS group will receive oral TCM treatment for 14 days, while the placebo group will receive a placebo for the same duration, both administered three times daily. The primary outcome measure is the cough relief rate on day 14. Secondary outcome measures include cough symptom scores (CSS), cough evaluation test (CET), TCM syndrome efficacy, Leicester Cough Questionnaire (LCQ) scores, cough cure rate, cough relief rate on day 7, time to cough cure, time to cough relief, and cough recurrence rate. Adverse events will be monitored and recorded throughout the study.

**Discussion:**

PIC is highly prevalent, yet current pharmacological treatment options remain limited. This trial is expected to generate evidence regarding the efficacy and safety of TCM in treating PIC and inform evidence-based recommendations for clinical practice.

**Clinical trial registration:**

https://itmctr.ccebtcm.org.cn/mgt/project/view/2038531670868623360, Identifier ITMCTR2025001210.

## 1 Introduction

Post-infectious cough (PIC) is characterized by a persistent cough that remains unresolved after the acute symptoms of a respiratory tract infection have subsided. It typically lasts for 3 to 8 weeks, while chest imaging remains normal (1). PIC is one of the most common types of chronic cough and stands as the leading cause of subacute cough (2). Adults typically experience 2–3 episodes of the common cold per year (3), with an incidence of influenza of 10.7% (4); moreover, the frequency of common colds tends to increase as immunity declines. Approximately 11 to 25% of adults develop PIC following a respiratory infection, a rate that can soar to 50% during epidemic seasons (5). Patients with a prior history of PIC are more likely to develop persistent cough after subsequent infections. The course of the disease significantly impairs patients’ work and quality of life, potentially leading to complications or misdiagnosis, which imposes unnecessary economic burdens and psychological distress. Current treatments for PIC primarily involve symptomatic relief using antitussives, antihistamines combined with decongestants. However, evidence supporting the efficacy of these regimens remains weak (1). Furthermore, these medications often entail unavoidable side effects such as somnolence and decreased appetite, and recurrence is common after discontinuation (6). Consequently, identifying an effective therapeutic approach with minimal side effects has become a focal point of current PIC research.

Traditional Chinese Medicine (TCM) offers distinct advantages in treating diseases through syndrome differentiation and holistic regulation. In the management of PIC, TCM has demonstrated significant efficacy in shortening cough duration and improving patients’ quality of life, characterized by high safety profiles and low recurrence rates (7). Our research group previously developed the Suhuang Zhike Capsule based on the “Wind-type Cough” theory proposed by National TCM Master Chao Enxiang. Composed of Herba *Ephedrae Herba* (Mahuang), *Cicadae Periostracum* (Chantui), *Perillae Folium* (Zisuye), *Perillae Fructus* (Zisuzi), *Schisandrae Chinensis Fructus* (Wuweizi), *Arctii Fructus* (Niubangzi), *Peucedani Radix* (Qianhu), *Eriobotryae Folium* (Pipaye), *Pheretima* (Dilong), this formula has shown remarkable therapeutic effects on PIC dominated by Wind-evil. It significantly improves the overall response rate and reduces cough severity (8). Consequently, it has been recommended in the Guidelines for Diagnosis and Treatment of Cough (9) as a therapeutic option. Previous pharmacological studies indicate that Suhuang Zhike Capsule improves inflammation in the airways and lung tissues (10–12). By regulating the neuro-immune network, it downregulates the levels of Substance P (SP) and Calcitonin Gene-Related Peptide (CGRP) in bronchoalveolar lavage fluid. Furthermore, it suppresses airway inflammation and the release of inflammatory mediators, thereby reducing cough receptor stimulation and decreasing cough frequency (13).

In recent years, changes in disease spectrum and modern lifestyle patterns have led to an increasing prevalence of internal dampness in patients with PIC, superimposed on the underlying pathogenesis of Wind-evil invading the lungs. Clinically, these patients present with features of latent wind invasion, such as coughing triggered by irritant odors or excessive talking, and pharyngeal itching, together with signs of dampness stagnation, including decreased appetite, abdominal distension, and a sticky oral sensation. Accordingly, based on the “Wind-type Cough” theory, our research group has proposed the syndrome termed Wind invasion with Dampness obstruction (hereafter abbreviated as Wind invasion-Dampness obstruction syndrome). The Shufeng Huashi (SFHS) formula was subsequently developed by modifying the original Suhuang Zhike Decoction to include additional dampness-resolving medicinal herbs. This study aims to evaluate the efficacy and safety of the SFHS formula in alleviating symptoms and improving outcomes for patients with PIC.

## 2 Methods

### 2.1 Study design

A randomized, double-blind, placebo-controlled, parallel-group, superiority clinical trial design will be employed. The study will be conducted across six hospitals in three regions of China: China-Japan Friendship Hospital (Beijing), the Third Affiliated Hospital of Beijing University of Chinese Medicine (Beijing), Wangjing Hospital of the China Academy of Chinese Medical Sciences (Beijing), Huairou Hospital of Beijing Traditional Chinese Medicine Hospital (Beijing), Wuqing District People’s Hospital (Tianjin), and the Affiliated Traditional Chinese Medicine Hospital of Southwest Medical University (Sichuan). Trial monitoring will be conducted on-site, with each study center receiving an initiation visit, a close-out visit, and two routine monitoring visits.

**Figure 1:**
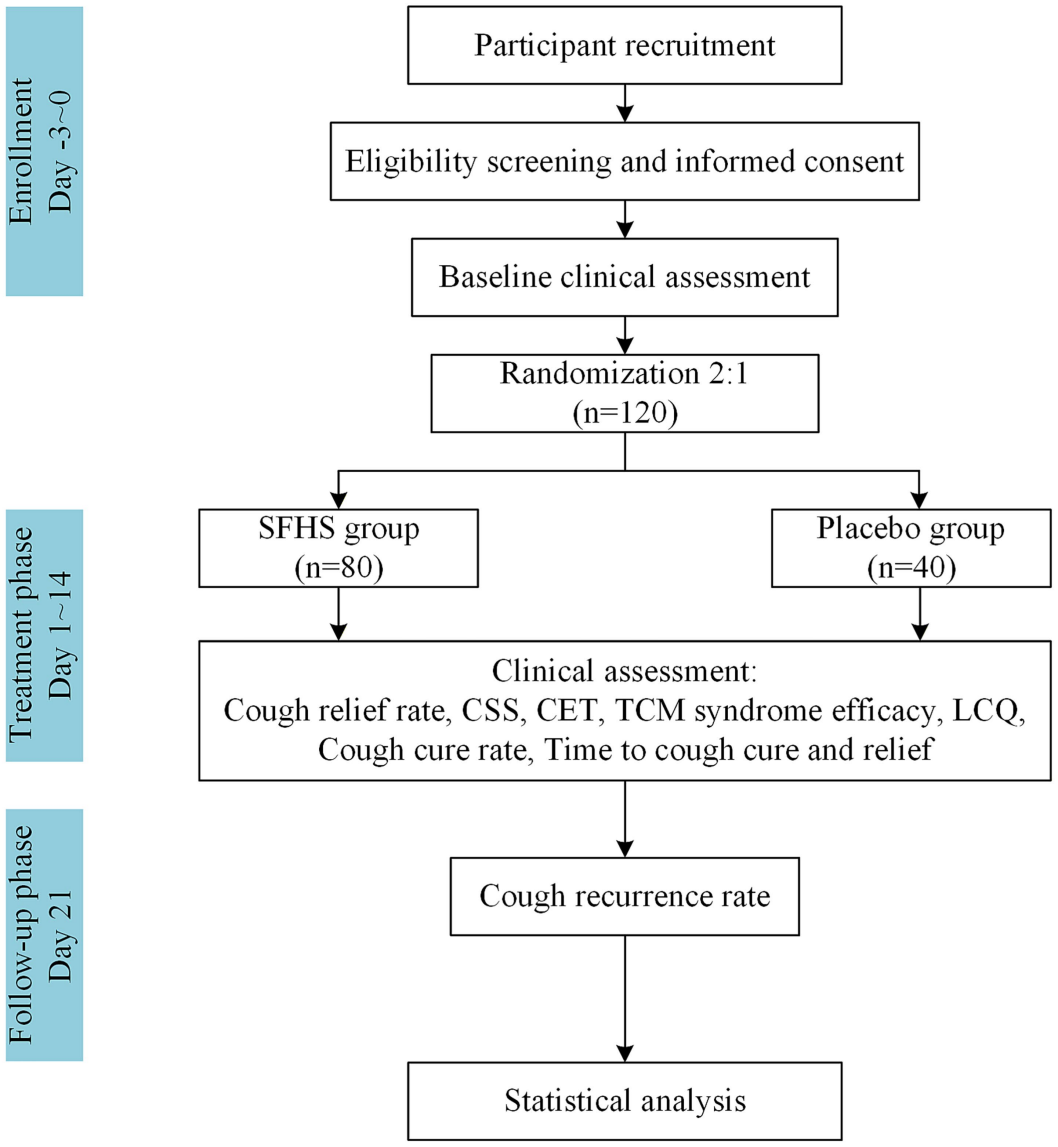
Study flowchart.

The overall study period spans from November 2024 to November 2027. The initial phase (November 2024 to October 2025) was devoted to protocol development and trial preparation, with no participant enrollment or screening conducted prior to the receipt of ethical approval and trial registration. Participant recruitment and intervention are scheduled between November 4, 2025, and November 30, 2027. The detailed study flow is illustrated in Figure 1. This protocol adheres to the SPIRIT 2025 guidelines and is conducted in accordance with the ethical principles outlined in the Declaration of Helsinki.

Ethical approval has been granted by the Clinical Research Ethics Committee of China-Japan Friendship Hospital (Approval No. 2025-KY-125-1). Any amendments to the trial protocol require submission to and re-approval by the Committee prior to implementation, and will be updated in the trial registry in a timely manner. This study is a joint research project sponsored by the China Association of Chinese Medicine and funded by Guangdong Efong Pharmaceutical Co., Ltd. Notably, the funder will not be involved in the study design, data collection and management, or data analysis. Participant recruitment for this trial is ongoing, and written informed consent has been obtained from all participants. The trial will be reported in compliance with the CONSORT guidelines and has been registered with the International Traditional Medicine Clinical Trial Registry (Identifier: ITMCTR2025001210; available at: https://itmctr.ccebtcm.org.cn/mgt/project/view/2038531670868623360). The detailed schedule for interventions and assessments is provided in Table 1.

**Table 1:** Study schedule.

Items	Screening (day −3 to Day 0)	Baseline (day 0)	Visit 1 (day 7 ± 1)	Visit 2 (day 14 ± 1)	Telephone follow-up (day 21 ± 1)
Written informed consent	√				
Collection of demographic data	√				
Medical history (past 3 months)	√				
Concomitant diseases and symptoms	√				
Vital signs	√		√	√	
Pulmonary auscultation	√		√	√	
Concomitant medications	√	√	√	√	√
CSS	√		Based on diary card records		√
CET	√		√	√	
TCM syndrome score	√		√	√	
LCQ	√			√	
Complete blood count	√			√	
Urinalysis	√			√	
Liver and renal Function tests	√			√	
12-Lead Electrocardiogram	√			√	
Chest X-ray or chest CT	√				
Pulmonary function test (including bronchodilator test)	√				
Serum pregnancy test (women of childbearing age)	√			√	
Adverse event recording		√	√	√	√
Randomization		√			
Dispensation of study medication		√			
Dispensation of diary card		√			
Return of study medication				√	
Return of diary card				√	
Compliance assessment			√	√	

### 2.2 Randomization and blinding

A block randomization method will be employed, with the randomization sequence generated by a statistician independent of the study conduct. Using an appropriate block length and a predefined random seed, a randomization sequence for 120 subjects will be generated via SAS 9.4 statistical software. Participants will be assigned to either the SHFS or placebo group at a 2:1 ratio. A randomization table will be generated, listing the drug kit numbers for assignment. Investigators will allocate these numbers to participants in the order they enroll, starting from the lowest number. A competitive enrollment mechanism will be implemented across all participating centers. This study is a double-blind trial; therefore, both investigators and outcome assessors will be blinded to the treatment allocation sequence. The investigational drug and the corresponding placebo will be identical in appearance and packaging. The blinding codes will be prepared in triplicate, sealed, and securely stored by the lead clinical research institution, the statistical unit, and the sponsor, respectively. Unauthorized access to blinding or emergency envelopes is strictly prohibited. Any premature unblinding of the codes will result in the disqualification of the clinical trial. Unblinding will be performed once, at the end of the trial or when necessary for safety reasons.

### 2.3 Participants

In this trial, PIC is defined according to the following criteria: (1) History of infection: A history of respiratory tract infection prior to the onset of cough. (2) Clinical manifestations: Presence of an irritant dry cough or a small amount of white, viscous sputum. (3) Duration: Cough typically persists for a period of 3 to 8 weeks. (4) Imaging findings: No significant abnormalities detected on chest X-ray or CT scan. (5) Differential diagnosis: Exclusion of cough caused by other underlying etiologies (e.g., cough-variant asthma, upper airway cough syndrome, or gastroesophageal reflux-induced cough).

The detailed diagnostic criteria for the Wind invasion-Dampness obstruction syndrome are provided in Table 2. The Cough Symptom Score (CSS) is divided into two components: daytime and nighttime scores. Based on the intensity and frequency of the cough, as well as the degree of its impact on the patient’s sleep and daily activities, the symptoms are categorized into four grades, scored from 0 to 3. The total CSS is defined as the sum of the daytime and nighttime scores (Table 3).

**Table 2:** Diagnostic criteria for Wind invasion-Dampness obstruction syndrome.

Category	Symptoms and signs
Primary symptoms	① Cough ② Throat itching or discomfort ③ Sudden onset or exacerbation triggered by temperature changes (cold or heat), irritant odors, or similar factors
Secondary symptoms	① Sticky sensation in the mouth ② Loss of appetite ③ Abdominal distension
Tongue signs	Pale tongue with white moist or white greasy coating
Pulse signs	Floating pulse, floating-moderate pulse, floating-soft pulse, floating-slippery pulse, floating-thin pulse, or normal pulse

The syndrome is diagnosed when the primary symptom is present, ≥2 secondary symptoms are met, together with corresponding tongue and pulse signs.

**Table 3:** Cough symptom score.

Daytime	Nighttime
□0 points: No cough	□0 points: No cough
□1 point: Occasional brief cough	□1 point: Brief cough at sleep onset or occasional nocturnal cough
□2 points: Frequent cough, mildly affecting daily activities	□2 points: Cough mildly affecting nighttime sleep
□3 points: Frequent cough, severely affecting daily activities	□3 points: Cough severely affecting nighttime sleep

#### 2.3.1 Inclusion criteria

Participants who meet all of the following criteria will be eligible for enrollment:

(1) Age: Aged 18–65 years (inclusive) at the time of informed consent.
(2) Western Medicine Diagnosis: Meeting the diagnostic criteria for PIC.
(3) TCM pattern identification: Wind invasion-Dampness obstruction syndrome.
(4) Symptom Severity: CSS of ≥ 3.
(5) Informed consent: Written informed consent was obtained.

#### 2.3.2 Exclusion criteria

Participants meeting any of the following criteria will be excluded from the trial:

(1) Other causes of cough: Cough caused by cough-variant asthma, upper airway cough syndrome, eosinophilic bronchitis, gastroesophageal reflux cough, atopic cough, chronic bronchitis, or other underlying etiologies; or persistent cough symptoms prior to the current infection.
(2) Severe pulmonary diseases: Presence of severe respiratory conditions such as chronic obstructive pulmonary disease, lung cancer, or pulmonary tuberculosis.
(3) Medication history: Use of angiotensin-converting enzyme inhibitors (ACEIs) within the past month.
(4) Smoking status: Current smokers or those who have quit smoking for less than 3 months.
(5) Comorbidities: Concurrent severe cardiovascular or cerebrovascular diseases, malignant tumors, hematological or hematopoietic system diseases, gastrointestinal diseases, or other severe or progressive systemic diseases; or severe mental illness or cognitive impairment rendering the participant unable or unwilling to cooperate.
(6) Hepatic or renal impairment: Alanine aminotransferase (ALT) or aspartate aminotransferase (AST) > 2 times the upper limit of normal (ULN); and/or serum creatinine (Cr) > 1.5 times the ULN.
(7) Abnormal laboratory findings: White blood cell (WBC) count <3.0 × 10^9^/L or >10.0 × 10^9^/L; and/or neutrophil percentage >80%.
(8) Body temperature: Axillary temperature ≥37.3 °C.
(9) Allergies: Known hypersensitivity to two or more substances, or allergy to the study drug or its ingredients.
(10) Substance abuse: History of long-term alcohol or drug abuse.
(11) Pregnancy and lactation: Women who are pregnant, breastfeeding, or planning to become pregnant.
(12) Other trials: Participation in other clinical trials within the past 3 months.
(13) Other concerns: Any other conditions that the investigator deems inappropriate for participation (e.g., poor compliance).

### 2.4 Interventions

Due to the lack of high-quality, strongly recommended pharmacological treatments for PIC, and considering its self-limiting nature where spontaneous remission often occurs, a placebo control was selected. The placebo is a simulated version of the SFHS granules. Study participants will be randomized into either the treatment group or the control group. The treatment group will receive SFHS granules, while the control group will receive SFHS simulators (placebo). Both will be administered orally, three times daily, for a continuous duration of 14 days. Both the SFHS granules and their corresponding placebos are manufactured by Guangdong Efong Pharmaceutical Co., Ltd. All medications were identical in appearance and packaging, and were labeled in a blinded manner. Each code corresponds to a unique drug allocation number in the randomization list. Each medication box contains 48 sachets (42 g per sachet) of either SFHS granules or the placebo, dispensed in a single batch upon completion of screening and enrollment. During subsequent follow-up visits, the number of used empty packages and remaining medications will be recorded to monitor compliance.

SFHS granules consist of *Ephedrae Herba* (Mahuang), *Armeniacae Semen Amarum* (Kuxingren), *Cicadae Periostracum* (Chantui), *Perillae Fructus* (Zisuzi), *Peucedani Radix* (Qianhu), *Arctii Fructus* (Niubangzi), *Stemonae Radix* (Baibu), *Acori Tatarinowii Rhizoma* (Shichangpu), *Magnoliae Officinalis Cortex* (Houpo), *Citri Reticulatae Pericarpium* (Chenpi), *Atractylodis Rhizoma* (Cangzhu), *Angelicae Sinensis Radix* (Danggui), and *Glycyrrhizae Radix et Rhizoma* (Gancao). The placebo contains starch, dextrin, and 5% SFHS granule components to ensure similarity to the SFHS granules in color, odor, taste, shape, and texture.

### 2.5 Concomitant treatment

Symptomatic treatments for comorbidities are permitted provided that they do not interfere with the evaluation of efficacy and safety. The generic name (or name of the therapy), dosage, rationale for use, frequency, and duration of such treatments are required to be meticulously documented in the medical records and electronic Case Report Forms (eCRF) for subsequent analysis and reporting during data synthesis. The use of other Western medicines, Traditional Chinese Medicine, or Chinese patent medicines with antitussive effects or therapeutic functions similar to the investigational drug is strictly prohibited.

### 2.6 Outcomes

#### 2.6.1 Primary outcome

The primary outcome measure is the cough relief rate after 14 days of treatment. Cough relief is defined as a total CSS (daytime + nighttime) ≤ 2, sustained for at least 48 h ± 2 h. Cough cure is defined as a total CSS (daytime + nighttime) ≤ 1, sustained for at least 48 h ± 2 h. The CSS will be recorded at baseline and daily throughout the study. The inter-group difference in the 14-day cough relief rate will be evaluated according to these diagnostic criteria.

#### 2.6.2 Secondary outcomes

(1) CSS: CSS will be recorded at baseline and daily. The differences in CSS between the two groups will be evaluated on day 7 and day 14.
(2) CET: CET scores will be recorded at baseline, day 7, and day 14 to evaluate the inter-group differences in CET scores at these time points.

The Cough Evaluation Test (CET) is a concise, patient-reported questionnaire comprising 5 items that assess key dimensions of cough impact, including daytime cough frequency, sleep disturbance, cough intensity, interference with work and daily activities, and cough-related anxiety. Each item is rated on a 5-point Likert scale from 1 (never) to 5 (always/frequently). The total score ranges from 5 to 25, with higher scores indicating more severe cough-related impairment.

(3) TCM Syndrome Efficacy: TCM syndrome scores will be recorded at baseline, day 7, and day 14 to assess the inter-group differences in both the scores and the overall clinical efficacy of TCM syndromes.

The TCM syndrome score is an instrument used to assess the severity of symptoms associated with the Wind invasion-Dampness obstruction syndrome. It comprises three major symptoms—cough, throat discomfort, and symptom aggravation triggered by cold, heat, or irritant odors—and three minor symptoms, including oral stickiness, poor appetite, and abdominal distension. Major symptoms are graded on a 4-level scale (absent, mild, moderate, and severe), scored 0, 2, 4, or 6 points respectively; minor symptoms are scored 0, 1, 2, or 3 points. Tongue appearance and pulse condition are additionally recorded to confirm the syndrome diagnosis. The total score is the sum of all individual symptom scores, with higher scores indicating greater TCM syndrome severity.

(4) LCQ: LCQ scores will be recorded at baseline and on day 14 to evaluate the inter-group differences in the improvement of quality of life.

The LCQ is a health-related quality of life instrument specifically designed to assess the impact of cough across three domains: physical (8 items), psychological (7 items), and social (4 items). Each of the 19 items is rated on a 7-point Likert scale ranging from 1 to 7. Domain scores are calculated as the mean of the constituent items (range 1–7), and the total score is the sum of the three domain scores, ranging from 3 to 21, with higher scores indicating better cough-related quality of life.

(5) Cough Cure and Relief Rates: Based on daily CSS records and predefined criteria, the inter-group differences in the cough cure rate (at day 7 and day 14) and the cough relief rate (at day 7) will be evaluated.
(6) Time to Cough Cure and Relief: Based on daily CSS records, the inter-group differences in the median time to reach cough cure and relief within the 14-day period will be analyzed.
(7) Cough Recurrence Rate: This is defined as the proportion of participants who achieved relief by the end of treatment but experienced a recurrence of cough during the 7-day post-treatment follow-up.

#### 2.6.3 Other outcomes

Routine laboratory assessments, including complete blood count (CBC), urinalysis, liver and renal function tests, and 12-lead electrocardiography (ECG), will be performed at baseline and on day 14 to monitor safety. Additionally, women of childbearing potential will undergo serum pregnancy tests at baseline and on day 14 to ensure the absence of pregnancy-related risks throughout the study period.

### 2.7 Sample size calculation

The sample size was estimated based on efficacy data from previous clinical trials. The cough relief rate for Suhuang Zhike Capsule was reported to be 87.75–93.33% (8, 14); thus, the expected relief rate for the Shufeng Huashi formula (π_T_) was estimated at 88%. The relief rate for the placebo, based on previous studies, ranged from 47.76–58.51% (15, 16); therefore, the expected relief rate for the placebo group (π_C_) was set at 48%. A 2:1 superiority design was employed, with a two-sided significance level of *α* = 0.05 and a power of 0.9 (1 − *β* = 0.9). A clinical superiority margin of 10% was considered. Using PASS 21 software for sample size estimation, the calculated requirement was 71 participants for the treatment group and 36 for the control group. Accounting for a 10% dropout rate and potential unforeseen data loss during trial execution, a total of 120 participants will be enrolled, with 80 in the treatment group and 40 in the control group. Participants will be recruited through outpatient clinics and advertisements.

### 2.8 Withdrawal criteria

#### 2.8.1 Investigator-initiated withdrawal

Investigator-initiated withdrawal refers to the removal of a randomized participant from the trial by the investigator due to circumstances that make it inappropriate for the participant to continue. Such participants will be withdrawn and provided with alternative treatments. Reasons include, but are not limited to: (1) Clinical changes: Development of severe comorbidities, complications, or specific physiological changes during the trial that, in the investigator’s judgment, render the participant unfit for continued participation. (2) Safety concerns: Occurrence of a serious adverse event (SAE) or other safety issues that necessitate the cessation of the trial intervention according to the investigator’s assessment. (3) Blinding violations: Cases involving premature unblinding or emergency unblinding must be withdrawn from the study.

#### 2.8.2 Participant-initiated withdrawal

Participants have the right to withdraw from the trial at any time. This includes: (1) Withdrawal of consent: The participant explicitly requests to terminate their participation in the trial. (2) Loss to follow-up: The participant does not explicitly withdraw but stops receiving the study medication or attending scheduled assessments and is subsequently lost to follow-up. All participants who discontinue the study early, except in cases of loss to follow-up, should complete subsequent visits according to the study schedule until the study endpoint.

### 2.9 Trial discontinuation and termination

Trial discontinuation, including temporary suspension or permanent termination, refers to the cessation of the entire clinical trial before its scheduled completion as per the protocol. The primary objectives of discontinuation are to protect the rights and welfare of participants, ensure the quality of the trial, and avoid unnecessary financial losses.

The clinical trial should be discontinued if any of the following situations occur: (1) Safety Issues: The occurrence of serious safety concerns where the investigator deems that the rights and well-being of the participants may be compromised. (2) Protocol or Implementation Deviations: The identification of major flaws in the clinical trial protocol or significant deviations during implementation that render the evaluation of the drug’s efficacy and/or safety unfeasible. (3) Sponsor Decisions: Request for discontinuation by the sponsor due to reasons such as funding constraints or administrative adjustments. (4) Regulatory Actions: Revocation of trial approval or orders to cease by pharmaceutical regulatory authorities.

### 2.10 Statistical analysis

Statistical analyses will be performed using SAS version 9.4 or a higher version.

The analysis populations are defined as follows: the Full Analysis Set (FAS) includes all randomized subjects who receive at least one administration of the investigational drug; the Per Protocol Set (PPS) includes all subjects in the FAS who comply with the study protocol, demonstrate good compliance, have complete primary efficacy endpoint data, do not use concomitant medications affecting efficacy evaluation, and have no major protocol deviations; the Safety Set (SS) includes all subjects who receive at least one administration of the study drug after randomization and have safety assessment data, and will be used as the safety analysis population.

Primary efficacy endpoint analysis: the primary endpoint is the cough relief rate on Day 14, defined as the proportion of subjects with a CSS total score ≤2 at the Day 14 visit and maintained for 48 h ± 2 h. Regardless of whether subjects experience transient relief during the treatment period, the final determination will be based on the CSS status at the Day 14 visit. This endpoint is a binary outcome and will be analyzed using the Cochran–Mantel–Haenszel (CMH) χ^2^ test stratified by center as the primary analysis model, separately in the FAS and PPS populations. Differences in relief rates between groups will be estimated using risk difference (RD), with point estimates, corresponding two-sided 95% confidence intervals, and *p* values reported. In addition, supplementary Logistic regression analyses will be performed with treatment group, center, and baseline CSS score as covariates to assess the robustness of the primary conclusions after covariate adjustment. For missing data of the primary endpoint, the Last Observation Carried Forward (LOCF) method will be used as the primary handling approach. To evaluate the robustness of the primary conclusions under assumptions regarding missing data, multiple imputation (MI) sensitivity analyses will be conducted under the Missing At Random (MAR) assumption, using baseline CSS score, treatment group, center, and all available post-baseline observations as imputation variables to impute missing values. The results obtained from both methods will be reported and compared simultaneously.

Secondary efficacy endpoint analysis: for quantitative variables, descriptive statistics will include number of cases (*n*), mean, standard deviation (SD), minimum, maximum, median, lower quartile (Q1), upper quartile (Q3), and 95% confidence interval (95%CI). Prior to analysis, normality tests will be performed for variable distributions: if the data conform to a normal distribution, inter-group comparisons will be conducted using the independent-samples *t* test, and within-group pre- and post-treatment comparisons will be conducted using the paired *t* test; if the data do not conform to a normal distribution, inter-group comparisons will be conducted using the Wilcoxon rank-sum test, and within-group pre- and post-treatment comparisons will be conducted using the Wilcoxon signed-rank test. For categorical variables, results will be described using frequencies and percentages. When considering the impact of center effects, binary and ordinal variables will be analyzed using the CMH χ^2^ test, and 95% confidence intervals for between-group risk differences will be provided for binary variables; when adjustment for baseline imbalance or other covariates is required, Logistic regression analysis will be used. For time-to-event variables, median time, lower and upper quartiles, and 95% confidence intervals will be described, Kaplan–Meier survival curves will be plotted, and inter-group comparisons will be performed using the log-rank test.

All statistical tests will be two-sided, and *p* < 0.05 will be considered statistically significant.

### 2.11 Safety assessment

All participants will undergo routine safety assessments at baseline and on Day 14, including CBC, urinalysis, liver and renal function tests, and 12-lead ECG. AEs (e.g., palpitations and gastrointestinal discomfort) related to medication use will be systematically monitored and recorded at each visit. The severity of AEs will be graded according to the Common Terminology Criteria for Adverse Events (CTCAE) v5.0. All AEs will be documented in the eCRF and reviewed by the Study Safety Committee. SAEs, including hospitalization, permanent disability, or life-threatening conditions, must be reported to the Institutional Ethics Committee within 24 h of occurrence. These events will be followed up continuously until resolution or stabilization.

### 2.12 Data storage and management

All study personnel are required to enter participant data online promptly after each visit. At the end of the trial, the eCRF for each participant will be exported as a PDF and archived electronically, with copies retained by both the sponsor and each participating clinical site for a period of 5 years following trial completion. Access to participants’ personal medical records is restricted to study personnel, monitors, and relevant administrative staff, all of whom must strictly adhere to applicable clinical trial regulations and principles. Trial data will be processed in an anonymized manner, with all personally identifiable information removed. All clinical trial records will be securely stored within the relevant departments of the clinical research center.

## 3 Discussion

The underlying pathophysiology of PIC remains incompletely understood, though current research suggests it is associated with airway mucosal damage, airway inflammatory response, airway hyperresponsiveness, and increased cough reflex sensitivity (17). Overall, the pathogenesis of PIC is considered a multifactorial process. In terms of treatment, corticosteroids, which are commonly used for anti-inflammatory purposes, have shown inconsistent efficacy in PIC; moreover, their long-term use can lead to adverse effects such as blood glucose fluctuations. Consequently, current guidelines no longer recommend corticosteroids for PIC. For viral-induced PIC, antibiotics are not recommended; furthermore, since PIC typically occurs after the acute phase of an upper respiratory tract infection, most patients do not meet the indications for antibiotic therapy, and their clinical use for PIC is rare (18). Montelukast sodium, a leukotriene receptor antagonist used to alleviate cough in asthma and prevent exercise-induced bronchospasm, has shown no statistically significant difference compared to placebo in clinical studies for PIC (19); thus, its use is also discouraged by guidelines. Currently, PIC treatment relies primarily on symptomatic therapies such as antitussives and antihistamine-decongestant combinations (9). However, evidence for these options is weak, and they often cause unavoidable side effects, including somnolence and loss of appetite, with a high tendency for recurrence after drug withdrawal. Given the high incidence of PIC and its persistent nature, finding a safe and effective treatment remains an urgent clinical challenge. This study aims to evaluate the clinical efficacy and safety of the SFHS formula for PIC through a randomized, double-blind, multicenter, placebo-controlled trial, with a one-week follow-up to demonstrate the advantages of TCM in providing sustained symptom relief.

In TCM, PIC is classified under the category of “Cough” (Ke Sou). In recent years, there has been an increasing number of clinical and experimental studies exploring the prevention and treatment of PIC through TCM (20–24). Meta-analyses have demonstrated that oral administration of Chinese herbal medicine is significantly effective in reducing cough symptom scores, enhancing the overall clinical response rate, and lowering the levels of airway inflammatory mediators, such as SP, interleukin-6, and neurokinin A (25). Given these therapeutic advantages, it is imperative to further investigate and establish safe and effective treatment regimens through rigorous research.

In recent years, shifts in disease epidemiology and modern lifestyles have led to a rising trend of the “Wind invasion-Dampness obstruction” syndrome in the PIC population. Patients with this pattern exhibit manifestations of latent Wind, such as coughing triggered by odors or excessive talking and pharyngeal itching, alongside characteristics of Dampness stagnation, including loss of appetite, abdominal distension, and a sticky sensation in the mouth. From the perspective of disease location, PIC is characterized by persistent irritant dry cough. The pathogenesis is rooted in the Lung’s failure to disperse and descend, leading to the upward reversal of Lung Qi. While the primary location is the Lung, the pathogenesis is closely linked to the viscera, particularly the “Lung-Spleen relationship.” Modern lifestyle factors—such as excessive consumption of cold drinks and greasy food, combined with a lack of physical activity—are primary causes of Spleen deficiency and internal Dampness. The Spleen, which “prefers dryness and loathes dampness,” is easily encumbered by Dampness, leading to impaired transport and transformation, which further causes fluid metabolic dysfunction and the accumulation of internal Dampness. When exogenous pathogens invade, they not only attack the Lung but also trigger internal Dampness. This Dampness ascends to affect the Lung, collectively inducing cough.

Based on the theory of “Wind Cough,” our research group proposed the “Wind invasion-Dampness obstruction” syndrome. By modifying the Suhuang Zhike decoction with additional dampness-resolving herbs, we developed the SFHS formula (26). *Ephedrae Herba* (Mahuang) serves as the monarch herb to disperse pathogenic factors in the lung, thereby relieving cough and asthma. *Armeniacae Semen Amarum* (Kuxingren) and *Stemonae Radix* (Baibu) act as minister herbs to suppress cough and resolve phlegm, while *Atractylodis Rhizoma* (Cangzhu) and *Magnoliae Officinalis Cortex* (Houpo) strengthen the spleen and eliminate dampness. *Perillae Fructus* (Zisuzi) and *Peucedani Radix* (Qianhu) are used as adjuvant herbs, which not only enhance the wind-dispelling effect of Mahuang but also promote the coordination of ascending and descending Lung qi. *Cicadae Periostracum* (Chantui), as the courier herb, further disperses wind pathogens and helps relieve throat itching. Since 2022, this formula has been used as an institutional formula in our hospital, with over 12,000 doses administered. This study aims to evaluate the efficacy and safety of the SFHS formula in alleviating clinical symptoms and reducing the recurrence rate of PIC.

The study employs a 2:1 allocation ratio (treatment vs. control) to minimize the exposure of participants to negative interventions (placebo), thereby enhancing recruitment feasibility and meeting ethical considerations. Regarding treatment duration, a review (unpublished data) of published RCTs on PIC conducted by our research team indicated that study durations predominantly range from 7 to 14 days. Accordingly, a 14-day continuous treatment period was selected, with assessments scheduled at days 7 and 14. This duration was considered sufficient to ensure adequate drug exposure for efficacy evaluation, while avoiding an excessively prolonged course that might compromise participant adherence. The day-7 visit additionally allows for assessment of early treatment response. To precisely evaluate the dynamic changes in cough severity and its impact on daily life, the cough relief rate was established as the primary outcome measure. Furthermore, a multidimensional evaluation framework—incorporating the CSS, CET, and LCQ—was implemented to overcome the inherent limitations of any single scoring system. From the perspective of TCM pattern identification and holism, the TCM Syndrome Score is utilized to assess the specific advantages of the SFHS formula in systemic symptom improvement. Finally, a one-week post-treatment follow-up was integrated to monitor symptom fluctuations and assess the recurrence rate after drug withdrawal, providing a comprehensive view of the treatment’s sustained efficacy.

This study has several strengths. First, the multicenter design enables recruitment of a broader and more diverse participant population, thereby reducing the selection bias associated with single-center studies and enhancing the generalizability of the findings. Second, the use of multiple cough assessment scales as outcome measures overcomes the limitations of any single tool and provides a multidimensional evaluation of subjective cough symptoms. Finally, to evaluate the durability of the therapeutic effect of SFHS, a one-week post-treatment follow-up period was incorporated (27, 28), informed by limitations identified in prior studies recommending surveillance of cough recurrence following treatment withdrawal (23, 24). This follow-up period allows for monitoring of symptom recurrence after treatment cessation and assessment of the sustained nature of the clinical response.

Despite the rigorous design, several limitations should be acknowledged. First, patients with PIC are prone to recurrent prolonged cough following subsequent respiratory tract infections. Although a one-week post-treatment follow-up was included, a longer observational period would provide more comprehensive insights into the long-term preventive effects of SFHS—for instance, tracking whether PIC recurs and assessing cough severity in the context of subsequent respiratory infections. Second, PIC occurs in the absence of active infection, so objective biomarkers are lacking, and reliance on patient-reported outcomes may introduce subjectivity. To reduce this, all investigators received standardized training to ensure consistent communication and interview techniques, maximizing the objectivity of cough frequency and severity scores. Third, blinding is essential for ensuring the objectivity and validity of a clinical trial. The present study did not include a formal assessment of blinding; future studies should incorporate blinding quality evaluation to further enhance methodological rigor. Fourth, compared with placebo preparations used in conventional Western medicine trials, the distinctive odor, taste, and color of TCM formulations present considerable challenges in placebo fabrication. To achieve adequate sensory matching with the active treatment, 5% of the active granule components were incorporated into the placebo. Although prior research has suggested that placebo preparations containing less than 10% of the active formula exhibit no appreciable pharmacological activity (29), the possibility that this 5% component exerted a residual therapeutic effect—and consequently led to an underestimation of the true efficacy of SFHS—cannot be entirely excluded. Finally, the present study employed a placebo-controlled design to establish the efficacy of the SFHS formula, but did not include a direct comparison with conventional antitussive medications. Future trials should incorporate active comparator arms to directly compare TCM interventions with standard-of-care antitussive treatments, thereby clarifying the relative therapeutic advantages of SFHS in the management of PIC.

In summary, this trial aims to evaluate the effects of the SFHS formula on cough symptoms, quality of life, and recurrence rates. By assessing both symptom relief and reduction in recurrence, the study will provide clinical evidence on the efficacy and safety of the SFHS formula in managing PIC.
